# Integrating Advanced Endoscopic Techniques and Confocal Microscopy for Early Detection of Extrahepatic Cholangiocarcinoma

**DOI:** 10.3390/cancers18091334

**Published:** 2026-04-22

**Authors:** Barbara Lattanzi, Francesco Covotta, Anna Crescenzi, Antonietta Lamazza, Francesco Maria Di Matteo, Domenico Alvaro, Vincenzo Cardinale

**Affiliations:** 1 Department of Gastroenterology and Endoscopy, Pertini Hospital, 00157 Rome, Italy; 2 Department of Translational and Precision Medicine, Sapienza University of Rome, 00185 Rome, Italy; francesco.covotta@uniroma1.it (F.C.); domenico.alvaro@uniroma1.it (D.A.); vincenzo.cardinale@uniroma1.it (V.C.); 3 Department of Radiological, Oncological and Anatomo-Pathological Sciences, Sapienza University of Rome, 00161 Rome, Italy; anna.crescenzi@uniroma1.it; 4 Department of Surgery, Sapienza University of Rome, 00185 Rome, Italy; antonietta.lamazza@uniroma1.it; 5 Digestive Endoscopy Unit, Campus Bio-Medico University Hospital, 00128 Rome, Italy; f.dimatteo@policlinicocampus.it; 6 Division of Gastroenterology, Department of Internal Medicine, Saint Louis University School of Medicine-SSM SLUCare, SLU Hospital, 1008 S. Spring, St. Louis, MO 63110, USA; 7 Department of Medicine, Harvard Medical School, 99 Brookline Ave, Boston, MA 02215, USA

**Keywords:** endoscopic ultrasound, extrahepatic cholangiocarcinoma, confocal microscopy, endoscopic retrograde cholangiopancreatography

## Abstract

Extrahepatic cholangiocarcinoma (eCCA) is a highly aggressive malignancy arising from the biliary epithelium, with surgical resection representing the only potentially curative treatment. The predominant periductal infiltrating growth pattern, characterized by subepithelial tumor spread and desmoplastic stromal reaction, severely limits the diagnostic sensitivity of conventional endoscopic sampling techniques, which primarily assess the luminal mucosal surface. This review provides a histomorphology-oriented diagnostic framework for indeterminate extrahepatic biliary strictures, integrating advanced endoscopic technologies with emerging optical diagnostic approaches. ERCP combined with cholangioscopy demonstrates superior sensitivity for perihilar strictures, while EUS-guided tissue acquisition shows higher diagnostic yield in distal cholangiocarcinoma, also providing locoregional staging. Advanced EUS technologies—including elastography, contrast harmonic EUS, and Detective Flow Imaging—further improve characterization of indeterminate strictures by evaluating tissue stiffness, microvascular architecture, and periductal infiltration. Ex vivo fluorescence confocal laser microscopy (FCM) enables real-time microscopic evaluation of biopsy specimens, reducing diagnostic turnaround time and minimizing inadequate sampling. A location-adapted diagnostic algorithm integrating cross-sectional imaging, ERCP, cholangioscopy, and EUS is proposed. An integrated, biology-informed endoscopic approach tailored to tumor location and ductal wall involvement may significantly improve early eCCA detection and guide patient selection for curative treatment.

## Simple Summary

Extrahepatic cholangiocarcinoma is an aggressive cancer of the bile ducts, and surgery offers the only real chance of cure. Unfortunately, it is often diagnosed late because the tumor tends to grow within the wall of the bile duct rather than forming a visible mass, making it difficult to detect with standard endoscopic sampling techniques that only reach the inner surface of the duct. In this review, we discuss how combining different advanced endoscopic tools—such as cholangioscopy, endoscopic ultrasound with tissue sampling, elastography, contrast-enhanced imaging, and microvascular flow imaging—can improve the early detection of this cancer. We also highlight the role of confocal microscopy, a new optical technology that allows pathologists to examine biopsy samples in real time, within minutes. We propose a diagnostic strategy tailored to tumor location, with the goal of identifying the disease earlier and helping more patients become eligible for curative treatment.

## 1. Introduction

Extrahepatic cholangiocarcinoma (eCCA) represents a biologically heterogeneous group of malignancies arising from the biliary epithelium and characterized by distinct anatomical, histopathological, and growth patterns that directly influence clinical presentation and diagnostic strategies [1,2]. According to their anatomical location, eCCA is traditionally classified into perihilar and distal tumors, which differ not only in surgical management but also in their diagnostic endoscopic approach [2]. Both entities frequently present clinically as indeterminate biliary strictures and establishing an accurate tissue diagnosis remains a major clinical challenge [1,3].

Recent advances in biliary pathology and molecular biology have significantly refined the understanding of cholangiocarcinoma pathogenesis [2,4,5]. Increasing evidence indicates that peribiliary gland cells represent an important cell of origin for large-duct cholangiocarcinoma, including intrahepatic large-duct cholangiocarcinoma, perihilar cholangiocarcinoma, and distal cholangiocarcinoma [6,7]. These epithelial progenitor niches located within the bile duct wall are also believed to give rise to precursor lesions such as intraductal papillary neoplasm of the bile duct, the biliary counterpart of pancreatic intraductal papillary mucinous neoplasms [4,5,7]. This biological framework highlights that neoplastic transformation frequently arises within, and extends along, the bile duct wall rather than being confined to the luminal epithelium.

Beyond anatomical classification, the histomorphological growth pattern of cholangiocarcinoma further influences disease behavior and diagnostic yield. Three principal macroscopic patterns are recognized: mass-forming, periductal infiltrating, and intraductal papillary growth [1]. Among these, the periductal infiltrating pattern is the most common growth pattern in extrahepatic cholangiocarcinoma and is characterized by extensive subepithelial tumor spread and a marked desmoplastic stromal reaction [6]. Consequently, substantial tumor infiltration of the bile duct wall may occur despite minimal mucosal abnormalities, thereby limiting the diagnostic sensitivity of endoscopic techniques that primarily sample the luminal surface.

Endoscopic retrograde cholangiopancreatography (ERCP) with brush cytology and intraductal biopsy remains the most widely used diagnostic approach for indeterminate biliary strictures [1,3]. However, the sensitivity of these techniques for detecting malignancy remains suboptimal [8], particularly in tumors characterized by predominant mural or subepithelial infiltration of the bile duct wall.

Peroral cholangioscopy has further expanded the endoscopic evaluation of biliary strictures by enabling direct visualization of the biliary mucosa and targeted tissue sampling. Nevertheless, because cholangioscopy primarily assesses the luminal mucosal surface, it may underestimate tumor spread within the ductal wall, particularly in lesions characterized by predominant subepithelial or stromal infiltration such as periductal infiltrating cholangiocarcinoma. Endoscopic ultrasound (EUS) has emerged as an important complementary modality in the evaluation of malignant biliary strictures, particularly in distal cholangiocarcinoma.

**Figure 1:**
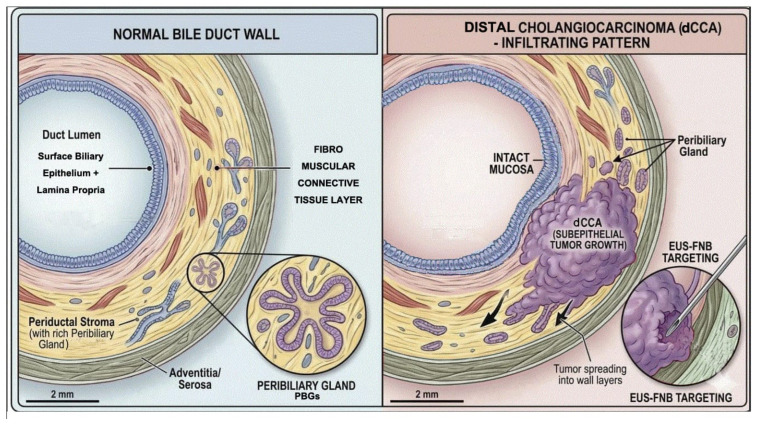
Biliary wall architecture and subepithelial infiltrating growth pattern of distal cholangiocarcinoma (dCCA). (**Left**) Cross-sectional anatomy of the normal extrahepatic bile duct, showing the surface biliary epithelium, fibromuscular connective tissue layer, periductal stroma with peribiliary glands (PBGs), and adventitia/serosa. (**Right**) In dCCA with periductal infiltrating pattern, the tumor grows within the deeper wall layers despite an intact luminal mucosa, illustrating the rationale for EUS-FNB transmural targeting over luminal sampling. Scale bar: 2 mm.

Taken together, these observations indicate that both anatomical location and tumor growth pattern critically influence the diagnostic performance of endoscopic techniques in suspected extrahepatic cholangiocarcinoma (Figure 1). Integrating knowledge of cellular origin, histomorphological growth patterns, and biliary wall architecture may therefore enable a more rational selection of endoscopic sampling strategies combining ERCP-based techniques, cholangioscopy-guided biopsy, and EUS-guided tissue acquisition according to the biological behavior of the lesion.

From an endoscopic perspective, this paradigm suggests that diagnostic strategies should be specifically designed to avoid overlooking neoplastic or malignant involvement of the biliary wall. Achieving this goal requires a broader conceptual approach to endoscopic training that integrates anatomical knowledge, histomorphology, pathology, and molecular correlates with clinical, radiological, and endoscopic findings. Such an integrative framework may support a new generation of endoscopists capable of interpreting biliary disease within a multidimensional biological context.

A similar conceptual evolution has already occurred in other complex gastrointestinal disorders. In inflammatory bowel disease, particularly Crohn’s disease, multidisciplinary and translational research centered on the concept of the intestinal barrier has generated a substantial body of knowledge that has transformed disease characterization and clinical management. An analogous model may be highly relevant for biliary diseases.

Primary sclerosing cholangitis provides a paradigmatic example in which chronic inflammation, progressive biliary remodeling, and field cancerization create a biological environment predisposing to cholangiocarcinoma [5,9]. In this setting, translational research is increasingly informing innovative diagnostic approaches, including contrast-enhanced imaging and advanced endoscopic ultrasound techniques aimed at detecting mural alterations of the bile duct wall and identifying early neoplastic transformation along the inflammation–dysplasia–carcinoma sequence.

The aim of this review was to evaluate the current state of the art and the available evidence in the literature regarding the diagnosis of extrahepatic cholangiocarcinoma, in order to develop a histomorphology-oriented diagnostic framework for indeterminate extrahepatic biliary strictures, integrating advanced endoscopic technologies with emerging optical and molecular diagnostic approaches.

## 2. Location-Adapted Diagnostic Strategy

The early diagnosis of eCCA is essential because surgical resection remains the only potentially curative treatment, yet most patients present with advanced disease due to the absence of specific early symptoms and the infiltrative growth pattern of these tumors [2]. In clinical practice, eCCA frequently presents as an indeterminate biliary stricture, rather than a well-defined mass, making differentiation from benign biliary conditions difficult and requiring a multimodal diagnostic approach [10].

Cross-sectional imaging represents the cornerstone of the initial diagnostic evaluation. Contrast-enhanced multiphasic computed tomography (CT) provides a comprehensive assessment of tumor presence, vascular involvement, lymph node enlargement, and distant metastases. Magnetic resonance imaging with magnetic resonance cholangiopancreatography (MRI/MRCP) offers superior visualization of the biliary tree and allows accurate delineation of the longitudinal extent of biliary strictures, which is essential for distinguishing distal from perihilar disease and for assessing surgical resectability [1]. However, although imaging plays a fundamental role in tumor detection and staging, histological confirmation remains necessary in most cases before initiating oncologic treatment, particularly when systemic therapy is planned [11].

ERCP remains a fundamental tool for tissue acquisition in extrahepatic cholangiocarcinoma through brush cytology and intraductal biopsy [1,3,12]. In addition to its diagnostic role, ERCP enables biliary drainage in patients with obstructive jaundice, which is essential for improving liver function and allowing subsequent surgical or systemic treatment. However, ERCP-based tissue acquisition is limited by relatively low sensitivity, particularly in infiltrative tumors, and negative cytology results do not exclude malignancy. According to the guidelines of the American Association for the Study of Liver Diseases (AASLD), the sensitivity of conventional cytology obtained via ERCP is limited (~43%), while specificity is high (~97%). The addition of techniques such as cholangioscopy and FISH can increase sensitivity [13,14,15]. Although cholangioscopy enables direct visualization and targeted biopsy, subgroup analyses suggest that its diagnostic yield in distal strictures may not consistently exceed fluoroscopic-guided sampling, likely due to technical instability and lesion characteristics. These findings emphasize that diagnostic performance is location-dependent [15,16]. These limitations highlight the need for complementary diagnostic modalities to improve diagnostic accuracy.

In this scenario, EUS has emerged as an important complementary tool in the diagnostic evaluation of eCCA. EUS provides high-resolution imaging of the distal bile duct and surrounding structures and allows tissue acquisition through fine-needle aspiration or biopsy (FNA/FNB). Several studies have demonstrated that EUS-guided tissue acquisition has high diagnostic accuracy in biliary strictures, particularly in distal lesions and in cases where ERCP sampling is inconclusive [17,18]. Reported sensitivity of EUS-FNA for distal cholangiocarcinoma ranges from approximately 79% to 89%, with specificity approaching 100% in many studies, highlighting its high diagnostic performance. In addition to its diagnostic role, EUS provides critical staging information. EUS allows evaluation of regional lymph nodes and vascular structures, facilitating accurate assessment of tumor extent and resectability [18,19,20]. EUS has demonstrated significant utility in identifying regional lymph node metastases that CT and MRI may fail to detect. A study of 157 patients with cholangiocarcinoma showed that EUS identified regional lymph nodes in 86% of cases compared with 47% using cross-sectional imaging (*p* < 0.001), with a sensitivity of 87.1% for lymph node metastases [21]. Specifically for perihilar cholangiocarcinoma, EUS-FNA identified lymph node metastases in 6.2% of cases and influenced the surgical decision in 1.5% of patients [22].

Despite its high diagnostic accuracy, the precise role of EUS within the diagnostic algorithm of cholangiocarcinoma remains a subject of ongoing debate. Some expert groups and reviews support early use of EUS, particularly in distal biliary strictures, owing to its higher sensitivity compared with ERCP-based cytology and its ability to provide simultaneous locoregional staging [23]. However, major European oncology and hepatology guidelines continue to position ERCP as the primary diagnostic modality in patients presenting with obstructive jaundice, particularly when biliary drainage is required [11]. The EASL guidelines emphasize cross-sectional imaging as the initial step, followed by ERCP for tissue acquisition and decompression when clinically indicated, with EUS considered complementary, especially for distal lesions or nodal staging [1].

An important element contributing to this debate is the potential risk of needle-tract seeding following EUS-guided tissue acquisition, particularly in perihilar cholangiocarcinoma. Evidence from transplant cohorts suggests an increased incidence of peritoneal metastases in patients who previously underwent transperitoneal biopsy of a hilar mass [23]. Although the absolute risk remains uncertain and data are limited to highly selected populations, this finding has influenced guideline recommendations, particularly in transplant-eligible patients. In contrast, in distal cholangiocarcinoma, where surgical treatment typically involves pancreaticoduodenectomy and the needle tract is frequently encompassed within the resection specimen, the theoretical oncologic risk appears substantially lower and EUS-guided tissue acquisition is more widely accepted [24,25]. Moreover, EUS-FNA has a higher sensitivity for detection of dCCA compared to pCCA [26].

Overall, the diagnosis of extrahepatic cholangiocarcinoma requires integration of cross-sectional imaging and endoscopic techniques to achieve optimal diagnostic accuracy while minimizing procedural risk. Contrast-enhanced CT and MRI/MRCP remain the initial diagnostic step, allowing assessment of tumor location, vascular involvement, and resectability. Tissue acquisition is mandatory for definitive diagnosis in most patients, particularly when systemic therapy is planned. ERCP maintains a central role in perihilar disease and in patients requiring biliary drainage, whereas EUS represents a highly valuable complementary modality, especially in distal cholangiocarcinoma, where it provides both tissue acquisition and locoregional staging. Moreover, EUS sensitivity is higher in comparison with cross-sectional imaging in identifying regional lymph nodes both in perihilar and distal CCA.

Given the complex diagnostic and therapeutic strategy required for extrahepatic cholangiocarcinoma, the management of this disease should be embedded within a structured multidisciplinary team (MDT) discussion, as recommended by all major international guidelines [1,10,11,24]. The core composition of the MDT typically includes hepatobiliary surgeons, gastroenterologists/interventional endoscopists, radiologists with expertise in hepatobiliary imaging, pathologists, medical oncologists and, in patients potentially eligible for liver transplantation, transplant hepatologists. Within this framework, the diagnostic process is best conceived as a closed loop in which each step feeds the next under shared decision-making. The MDT discussion should be activated after completion of cross-sectional imaging, when the first elements regarding tumor location, longitudinal extent, vascular involvement, and provisional resectability become available, and reconvened after tissue acquisition and at the final treatment decision, in order to integrate radiological, endoscopic, and pathological findings into a coherent therapeutic decision. Within this process, the radiologist defines the anatomical extent and provisional resectability of the lesion, the hepatobiliary surgeon confirms surgical operability and the indication for resection or transplantation, the interventional endoscopist selects the most appropriate sampling strategy according to tumor location and ductal wall involvement and ensures adequate biliary drainage when required, the pathologist provides definitive histological characterization and ancillary molecular analyses, and the medical oncologist defines the systemic treatment strategy in unresectable or metastatic disease. This integrated, closed-loop approach ensures that diagnostic information is translated into therapeutic decisions in a coherent and timely manner, and represents the optimal context within which the location-adapted diagnostic strategy proposed in this review should be applied.

**Figure 2:**
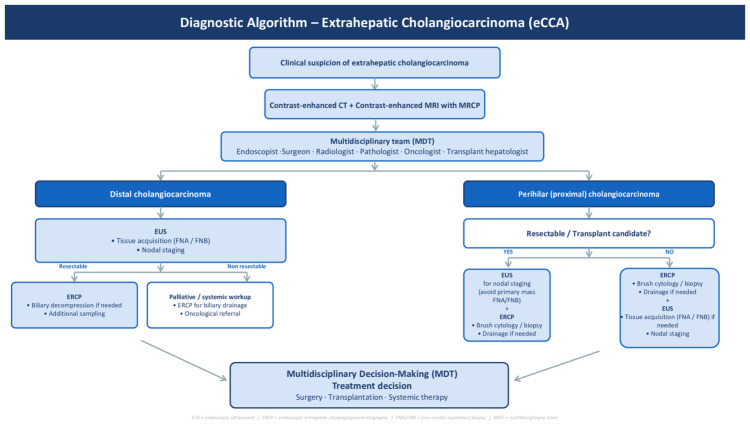
Location-adapted diagnostic algorithm for extrahepatic cholangiocarcinoma. Proposed location-adapted diagnostic algorithm for extrahepatic cholangiocarcinoma (eCCA). The algorithm stratifies the diagnostic approach based on tumor location (perihilar vs. distal), integrating cross-sectional imaging, ERCP with cholangioscopy, and EUS-guided tissue acquisition according to the biological behavior and anatomical characteristics of the lesion.

The comparative diagnostic performance of these modalities according to tumor location is summarized in Table 1, which provides a practical framework for selecting the most appropriate sampling strategy in perihilar versus distal eCCA. The integration of imaging, ERCP, and EUS within a multidisciplinary framework forms the basis of a location-adapted diagnostic strategy and underpins the diagnostic algorithm proposed below (Figure 2).

## 3. Advanced Endoscopic Techniques: From Indeterminate Strictures to Targeted Diagnosis

The diagnostic challenge of eCCA lies not only in tumor detection but also in the accurate characterization of indeterminate biliary strictures and the acquisition of adequate tissue samples. Conventional fluoroscopic-guided sampling and standard EUS imaging, although fundamental components of the diagnostic work-up, may be insufficient in the presence of subtle mucosal abnormalities or infiltrative submucosal tumor growth. These limitations have driven the development of advanced endoscopic technologies aimed at improving direct visualization and targeted tissue acquisition.

### 3.1. Cholangioscopy

Cholangioscopy consists of direct intraductal endoscopic visualization of the biliary tree performed during ERCP, allowing real-time assessment of mucosal patterns and targeted biopsy of suspicious areas. By overcoming the limitations of blind fluoroscopic sampling, cholangioscopy significantly enhances diagnostic performance, particularly in indeterminate strictures. Cholangioscopy-guided biopsies have demonstrated sensitivities ranging from approximately 60–75%, with consistently high specificity (93–97%), clearly outperforming conventional brush cytology alone [13,14,15]. Both the American Society for Gastrointestinal Endoscopy and European guidelines recommend the use of cholangioscopy-directed biopsy in patients with biliary strictures of undetermined etiology, especially after nondiagnostic ERCP sampling and in centers with adequate expertise [1,15].

Beyond targeted tissue sampling, direct intraductal visualization allows the endoscopist to recognize specific mucosal and vascular features that are considered suggestive of malignancy. The cholangioscopic findings most consistently associated with malignant biliary strictures include irregular and tortuous neovascularization (the so-called “tumor vessels”), intraductal nodular or papillary projections, irregular or infiltrative mucosal patterns, and increased mucosal friability with spontaneous bleeding. Several classification systems have been proposed to standardize the cholangioscopic interpretation of indeterminate biliary strictures and to improve interobserver agreement, including the classification proposed by Robles-Medranda et al. [27] and the more recent Monaco classification developed by Sethi et al. [28], although their diagnostic accuracy remains operator- and experience-dependent. The integration of these visual criteria with targeted biopsy sampling represents the main advantage of cholangioscopy over conventional fluoroscopic-guided techniques in the evaluation of indeterminate biliary strictures.

Importantly, diagnostic performance differs according to tumor location. Available evidence indicates that cholangioscopy-guided biopsy achieves higher sensitivity in proximal (hilar) strictures compared with distal lesions, with reported sensitivities around 67% for proximal tumors versus approximately 50% for distal ones [15]. Prospective data further support improved accessibility and higher rates of successful targeted biopsies in perihilar tumors [16]. These differences likely reflect anatomical accessibility, the intraductal growth pattern of perihilar cholangiocarcinoma, and the greater technical difficulty in stabilizing the cholangioscope within distal strictures. For these reasons, ERCP combined with cholangioscopy represents a particularly rational first-line endoscopic strategy in suspected proximal extrahepatic cholangiocarcinoma, where intraductal visualization and targeted sampling offer clear diagnostic advantages.

In contrast, in distal cholangiocarcinoma, where cholangioscopy appears less sensitive and where transperitoneal seeding is less of a concern compared with perihilar tumors considered for transplantation, EUS assumes a more central diagnostic role.

Moreover, because cholangioscopy primarily assesses the luminal mucosal surface, it may underestimate tumor spread within the ductal wall, particularly in lesions characterized by predominant subepithelial or stromal infiltration, such as periductal infiltrating cholangiocarcinoma. This limitation may contribute to false-negative biopsy results when neoplastic involvement predominantly affects the ductal wall rather than the surface epithelium.

Unlike cholangioscopy, which primarily evaluates the luminal surface of the bile duct, EUS-based techniques provide information about mural and periductal structures, making them particularly suitable for detecting subepithelial tumor infiltration.

### 3.2. Advanced EUS-Based Techniques

The integration of advanced technologies in EUS, such as elastography, Contrast Harmonic EUS, and Detective Flow Imaging, is revolutionizing the diagnostics of biliopancreatic lesions, offering promising tools even for the early diagnosis of cholangiocarcinoma [37].

**Assessing Tissue Stiffness: Elastography.** A significant leap forward in diagnostic precision is guaranteed by EUS-guided elastography, which provides direct information on tissue “hardness” or elasticity in real time, without the need to inject any contrast medium. Neoplastic tissues, frequently characterized by an intense desmoplastic reaction and cellular fibrosis, are significantly stiffer compared to healthy or inflammatory soft tissues. Elastography is divided into two fundamental approaches: qualitative (strain elastography) and quantitative (shear wave elastography). Strain elastography evaluates the axial deformation of the tissue in response to slight compressions directly induced by the ultrasound probe, with dedicated software generating a color map where hard tissues (suspicious for malignancy) appear in blue, while more elastic and healthy tissues are represented in red or green. The calculation of the strain ratio has shown extremely high sensitivities in identifying malignant masses compared to inflammatory ones [37]. More recently, shear wave elastography introduced purely quantitative measurement, emitting a high-intensity pulse that generates transverse shear waves whose propagation speed is rigorously measured and converted into kilopascals (kPa) or meters per second [38]. In the clinical context of a biliary stricture, quantitative elastography could measure the exact degree of stiffness of the affected duct wall, directing the physician toward extremely targeted biopsy sampling.

**The Role of Contrast Media: Contrast Harmonic EUS (CH-EUS).** Although traditional radiological imaging modalities, such as CT and MRI, are excellent for disease staging and overall vascular assessment, they face significant limitations in the early diagnosis of small biliopancreatic lesions [39]. CT scans exhibit reduced sensitivity for lesions under 2 cm, dropping to 71% in some comparative studies [40], compared to 98% for standard EUS [41]. Similarly, MRI provides superior soft-tissue contrast but is highly susceptible to motion artifacts and requires lengthy acquisition times [42], limiting its sensitivity for small masses to 84% [29].

In this context, the introduction of Contrast Harmonic EUS (CH-EUS) offers an interesting perspective to mitigate these limitations. This technique utilizes second-generation ultrasound contrast agents made of inert gas microbubbles encapsulated in a resistant shell. When insonated by ultrasound waves emitted at a low mechanical index, these microbubbles vibrate and produce a strong acoustic signal, allowing for excellent visualization of the microvasculature and eliminating typical Doppler artifacts [29,30]. In studies on solid lesions, CH-EUS has demonstrated the ability to detect lesions as small as 15 mm [29], distinguishing adenocarcinoma (typically associated with a “hypo-enhancement” pattern) with a sensitivity of up to 96% and a specificity of up to 94% [31].

Furthermore, translating the solid clinical evidence obtained in the hepatobiliary tract and gallbladder, CH-EUS proves to be a crucial tool for detecting highly suspicious findings, such as vascularization within the lesion or signs of deep invasion. The identification of intratumoral irregular vascularization has demonstrated a sensitivity of 94% and a specificity of 93% for malignancy in this area [32]. This technique has also proven significantly superior to standard ultrasound (B-mode) in differentiating true focal lesions from benign obstructions like sludge plugs, reaching a diagnostic accuracy of 99% [33]. Applying these premises to the evaluation of a suspicious biliary stricture, mapping the microvascular architecture could provide a valuable aid to the clinician in orienting the suspicion toward an invasive cholangiocarcinoma (characterized by aberrant and irregular microvessels) rather than a simple accumulation of thick bile material or an inflammatory reaction.

**Detective Flow Imaging (DFI): Beyond the Limits of Doppler.** Another recent digital technological development in the field of microvascular assessment is Detective Flow Imaging (DFI). Historically, visualizing minute and extremely slow-flowing blood vessels within tumors has been hindered by motion artifacts caused by breathing or cardiac pulsations [34]. DFI is an algorithmic technology that allows for the extraction of only the signal from very low-speed blood flow, actively canceling motion artifacts based on signal intensity. Operating at a high frame rate, it has proven significantly superior in speed and clarity compared to previous technologies like eFLOW, reaching an accuracy of 88% for the diagnosis of malignant lesions [35].

In recent clinical studies on solid lesions, DFI was able to detect blood vessels within the lesion in 96% of cases. Patterns of “hypovascularized” lesions, characterized by vessels distributed in the peritumoral area or with a spotty morphology, have been validated as suggestive criteria for malignancy [33]. Applied to the hepatobiliary setting, DFI has also demonstrated superiority over eFLOW in discriminating between true solid lesions and biliary sludge, identifying irregular vessel patterns as a significant predictor of malignancy [34]. Furthermore, detecting these microscopic vascular irregularities within the bile duct wall could not only raise a strong diagnostic suspicion but also indicate to the endosonographer the ideal zones to avoid large vessels during fine-needle biopsy (EUS-FNB), improving procedural safety.

The reported diagnostic performance, main advantages, and current limitations of these advanced EUS-based technologies are summarized in Table 2.

## 4. Confocal Microscopy

While advanced endoscopic imaging has substantially improved macroscopic lesion characterization and targeted tissue acquisition, further refinement of diagnostic accuracy increasingly relies on technologies capable of providing real-time microscopic assessment. In this evolving landscape, integrating enhanced endoscopic visualization with optical histology represents a critical step toward earlier and more precise detection of extrahepatic cholangiocarcinoma.

New optical imaging technologies represent a cutting-edge advancement in the diagnostic evaluation of histological and cytological specimens. Ex vivo fluorescent confocal laser microscopy (FCM) is referred to as “instant digital microscopy” because it enables immediate digital pathological assessment of fresh, unfixed tissue, thereby bypassing the preparation of conventional histological glass slides. FCM generates digital microscopic images closely resembling hematoxylin and eosin-stained sections directly from native biological specimens, without inducing structural damage or distortion. Importantly, the tissue remains fully available for subsequent formalin fixation and ancillary investigations. This technology employs two laser sources to produce high-resolution digital images through photon interactions with both labeled and unlabeled tissue components.

The protocol requires only a few drops of acridine orange on the tissue to enhance nuclear visualization. Initially developed to facilitate rapid intraoperative assessment of surgical resection margins, FCM is now increasingly adopted for the immediate evaluation of small tissue samples, including image-guided core needle biopsies and endoscopic biopsy specimens. The acquisition of real-time digital images enables prompt evaluation of specimen adequacy, immediate diagnostic interpretation, even from remote settings, and digital sharing among pathologists.

The entire workflow requires approximately five minutes, while preserving tissue integrity. After FCM imaging, the specimen can be retrieved intact and processed according to routine formalin fixation and paraffin embedding (FFPE) protocols for permanent histology and ancillary molecular or immunohistochemical analyses.

The morphological features assessable by FCM closely parallel those evaluated on conventional hematoxylin and eosin sections. The use of acridine orange enhances nuclear contrast and allows direct assessment of nuclear size, shape, and chromatin distribution, as well as of the nuclear-to-cytoplasmic ratio. Tissue architecture, glandular organization, and the relationship between epithelial structures and the surrounding stroma are simultaneously preserved, enabling the pathologist to recognize the loss of regular epithelial architecture, the presence of solid or acinar growth patterns, and the disruption of the normal ductal lining that characterize biliary malignancies. These features can be appreciated in real time on fresh, unfixed tissue, providing a morphological readout that is directly comparable to standard histology and that can guide the immediate decision on specimen adequacy.

Recent investigations on pancreatic EUS-FNB specimens from solid pancreatic lesions have demonstrated that FCM reduces diagnostic turnaround time and ensures adequate sampling, thereby minimizing the need for additional biopsy passes [36,43]. Despite its expanding clinical application, extrahepatic cholangiocarcinoma represents a particularly challenging target for EUS/FNAB due to its anatomical location and intrinsic histopathological features, and no data are currently available regarding the use of FCM in this specific setting. Accordingly, the optimal timing for FCM evaluation within the diagnostic workflow, its complementary relationship with conventional pathology, and standardized operational procedures—including dedicated working pipelines, acquisition parameters, and validated interpretation criteria—have not yet been defined for extrahepatic cholangiocarcinoma and represent aspects that will require dedicated validation studies in the near future.

In this context, FCM may ensure specimen adequacy and shorten diagnostic timelines. Digital real-time images provide high-resolution visualization of tissue architecture, as well as cellular and subcellular details, enabling accurate morphological assessment by pathologists. Results reported in other echoendoscopic procedures underscore the clinical relevance of this approach in shortening the time from biopsy to therapeutic decision-making in a high-risk patient population and highlight the value of immediate digital real-time sample evaluation in the endoscopy room to prevent repeat procedures due to inadequate sampling [44]. This strategy has the potential to standardize and accelerate the diagnostic workflow for cholangiocarcinoma, ultimately improving patient management and clinical outcomes.

**Figure 3:**
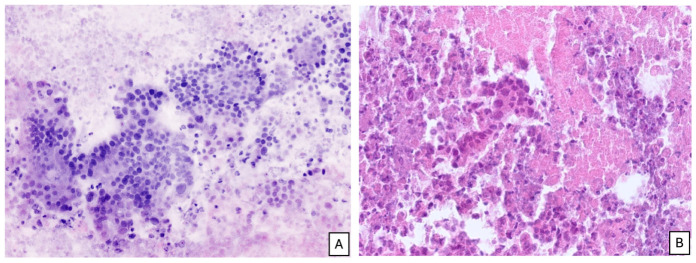
Comparison between instant digital imaging and conventional permanent paraffin section in a case of EUS-FNA from pancreatic solid lesion. Neoplastic cells are easily identified in the necrotic background both in the native digital image (**A**) and in the permanent histology (**B**).

A representative example of this approach is shown in Figure 3, which illustrates the side-by-side morphological correspondence between an ex vivo fluorescence confocal microscopy image of an EUS-guided fine-needle biopsy specimen and the corresponding conventional hematoxylin and eosin section obtained from the same specimen after standard formalin fixation and paraffin embedding. As no published data are currently available on the application of FCM to biliary EUS-FNB specimens, the case shown is provided as a proof-of-concept of the morphological correlation that can be achieved between real-time digital pathology and conventional histology in this clinical setting.

As a complementary molecular approach, fluorescence in situ hybridization (FISH) can be applied to cytologic or biopsy specimens obtained during ERCP or EUS. By detecting chromosomal abnormalities such as polysomy in biliary epithelial cells, FISH enhances the diagnostic sensitivity of conventional cytology, particularly in indeterminate strictures with high clinical suspicion. Although not a standalone diagnostic tool, FISH may increase diagnostic confidence when standard sampling yields inconclusive results [8].

## 5. Conclusions

Extrahepatic cholangiocarcinoma remains one of the most challenging malignancies of the hepatobiliary system, largely because many tumors grow along the bile duct wall rather than forming well-defined intraluminal masses. This biological behavior explains why indeterminate biliary strictures frequently yield inconclusive results with conventional sampling techniques.

Increasing knowledge of tumor biology and growth patterns suggests that diagnostic strategies should be tailored according to both tumor location and the depth of ductal wall involvement. ERCP-based techniques, particularly when combined with cholangioscopy, play a central role in the evaluation of perihilar strictures, where direct intraductal visualization and targeted biopsies can improve diagnostic yield. Conversely, endoscopic ultrasound has emerged as a key modality in distal cholangiocarcinoma, providing high-resolution imaging, tissue acquisition, and locoregional staging.

Recent technological advances—including cholangioscopy, contrast-enhanced EUS, elastography, and microvascular imaging—are progressively improving the characterization of indeterminate strictures and guiding more precise tissue sampling. In parallel, emerging optical pathology approaches such as confocal microscopy may enable real-time microscopic evaluation of biopsy specimens, bridging the gap between endoscopy and histopathology.

Future diagnostic strategies will likely rely on the integration of anatomical knowledge, tumor growth patterns, advanced endoscopic imaging, and molecular diagnostics. Such an integrated approach may facilitate earlier detection of cholangiocarcinoma and ultimately improve patient selection for curative treatment. The integration of advanced endoscopic imaging with real-time optical pathology may represent a key step toward a more precise and biologically informed diagnostic framework for extrahepatic cholangiocarcinoma.

## Figures and Tables

**Table 1:** Comparative role and diagnostic performance of endoscopic and ancillary modalities for the diagnosis of extrahepatic cholangiocarcinoma (eCCA) according to tumor location (perihilar vs. distal).

Diagnostic Modality	Role in Perihilar eCCA	Role in Distal eCCA	Diagnostic Performance	Key Limitations
ERCP + brush cytology	First-line sampling during drainage Central when decompression needed	First-line sampling during drainage Often complemented by EUS	Sensitivity ~43% Specificity ~97% [13]	Low sensitivity in infiltrative tumors Negative cytology does not exclude malignancy [8,13]
ERCP + intraductal forceps biopsy	Complementary to brushing Better if combined with cholangioscopy	Complementary to brushing Yield limited by stricture access	Suboptimal alone Improved with cholangioscopy/FISH [8,13,14,15]	Samples luminal mucosa only May miss subepithelial infiltration
Cholangioscopy-guided biopsy	Preferred endoscopic strategy Direct intraductal visualization + targeted biopsy	Less effective Cholangioscope instability in distal strictures	Proximal ~67% vs. distal ~50% Overall 60–75%; specificity 93–97% [14,15,16]	Assesses luminal surface only May underestimate mural infiltration
EUS-FNA/FNB	Complementary role Debated due to needle-tract seeding risk in transplant candidates	Central diagnostic role Provides tissue + locoregional staging	~79–89% for distal eCCA Specificity approaching 100%; higher in distal than perihilar	Seeding risk in perihilar [23] Lower concern in distal (tract within pancreaticoduodenectomy)
Advanced EUS imaging (elastography, CH-EUS, DFI)	Ancillary; characterizes ductal wall stiffness and microvasculature	Ancillary; maps stiffness, microvessels, periductal infiltration prior to sampling	Elastography: malignant-inflammatory discrimination [27,28] CH-EUS: sensitivity up to 94%, specificity up to 93%; accuracy up to 99% [29,30,31,32] DFI: microvessel detection 96%; accuracy 88% [33,34]	Mostly validated in pancreas/gallbladder Data on eCCA limited Operator- and equipment-dependent
FISH (ancillary)	Increases sensitivity of brush cytology Especially in PSC-related strictures	Increases sensitivity when conventional sampling inconclusive	Improves cytology sensitivity when added to ERCP sampling [8]	Not stand-alone Adjunct to increase confidence in indeterminate strictures
Ex vivo fluorescence confocal microscopy (FCM)	Emerging tool Real-time adequacy and preliminary interpretation	Emerging tool Promising for real-time EUS-FNB evaluation	Reduces turnaround time in pancreatic EUS-FNB Data on eCCA not yet available [34,35,36]	No standardized biliary protocols Tissue preserved for FFPE and ancillary analyses

ERCP, endoscopic retrograde cholangiopancreatography; EUS, endoscopic ultrasound; FNA, fine-needle aspiration; FNB, fine-needle biopsy; CH-EUS, contrast-harmonic EUS; DFI, Detective Flow Imaging; FISH, fluorescence in situ hybridization; FCM, fluorescence confocal microscopy; FFPE, formalin-fixed paraffin-embedded; PSC, primary sclerosing cholangitis; eCCA, extrahepatic cholangiocarcinoma.

**Table 2:** Comparative diagnostic performance, advantages, and current limitations of advanced endoscopic ultrasound technologies for the characterization of indeterminate biliary strictures.

Advanced EUS Technology	Reported Sensitivity	Reported Specificity	Main Advantages	Current Limitations
Elastography (strain and shear-wave)	Up to 100% for malignancy in solid pancreatic masses 100% in adenocarcinoma vs. inflammatory masses [37]	Up to 92.9% for malignancy 96.3% for adenocarcinoma vs. inflammatory masses Accuracy 97.7%, AUC 0.983 [37]	Real-time, contrast-free stiffness assessment Detects desmoplastic reaction Quantitative with shear-wave May guide biopsy toward stiffest areas	Mostly validated in pancreas Operator- and equipment-dependent No standardized cut-offs for biliary strictures Affected by stents and inflammation
Contrast Harmonic EUS (CH-EUS)	Up to 94% via irregular intratumoral vascularization [31] Up to 96% for small solid pancreaticobiliary lesions [30]	Up to 93% for malignancy [31] Up to 94% in pancreatic adenocarcinoma [30] Accuracy up to 99% vs. biliary sludge [32]	Microvascular visualization without Doppler artifacts Superior to B-mode vs. sludge/inflammation Detects lesions ≥15 mm [29] Directs FNB to viable tumor areas	Requires IV contrast agents Operator- and center-dependent Data on eCCA still limited Less informative in fibrotic/stented strictures
Detective Flow Imaging (DFI)	99% for pancreatic cancer (hypovascular/peritumoral/spotty criteria) Intralesional microvessels in 96% vs. 27% with eFLOW [33]	43% for pancreatic cancer Accuracy 88% [34] Low specificity reflects sensitivity-maximizing criteria	Contrast-free, high-frame-rate imaging Cancels motion artifacts Superior to Doppler and eFLOW Discriminates solid lesions from sludge May guide FNB to avoid large vessels	Limited validation in eCCA Mostly from pancreas/gallbladder Modest specificity (43%) due to overlap with mass-forming pancreatitis Equipment-dependent No standardized biliary criteria

EUS, endoscopic ultrasound; CH-EUS, contrast harmonic endoscopic ultrasound; DFI, Detective Flow Imaging; FNB, fine-needle biopsy; eCCA, extrahepatic cholangiocarcinoma; AUC, area under the ROC curve.

## Data Availability

No new data were created or analyzed in this study. Data sharing is not applicable to this article.
